# Supplementary material for: Heat adaptation of phage T7 under an extended genetic code
Source: Virus Evol. 2021 Dec 1;7(2):veab100. doi: 10.1093/ve/veab100 (PMC8923235; doi:10.1093/ve/veab100)
Supplement: veab100_Supp [file veab100_supp.zip › Sequencing_Analysis.zip!/Sequencing_Analysis/A3/evidence/SNP_6.html]

BRESEQ :: Evidence


| Predicted mutation | | | | | | |
| --- | --- | --- | --- | --- | --- | --- |
| evidence | seq id | position | mutation | annotation | gene | description |
| RA | NC\_001604.1 | 24,161 | A→C | intergenic (–/–) | – / – | –/– |

| Read alignment evidence... | | | | | | | | | | | |
| --- | --- | --- | --- | --- | --- | --- | --- | --- | --- | --- | --- |
|  | seq id | position | | ref | new | freq | score (cons/poly) | reads | annotation | genes | product |
| \* | NC\_001604.1 | 24,161 | 0 | A | C | 99.5% | 3904.0 / NA | 1293 | intergenic (–/–) | *–/–* | –/– |
| Reads supporting (aligned to +/- strand):  **ref** base A (3/4);  **new** base C (692/587);  **total** (698/595) | | | | | | | | | | | |
| Fisher's exact test for biased strand distribution *p*-value = 7.09e-01 | | | | | | | | | | | |
| Kolmogorov-Smirnov test that lower quality scores support variant *p*-value = 1.00e+00 | | | | | | | | | | | |

Only 100 of 1293 total aligned reads displayed.  

`GTACTGTTAAGCTGCGTGACTTGGCTCTGGAGCGCGCTCGCCGTGCTAACTTCCAAGCGGACCAGATTATCGCTAAGTACGCAATGGGCCACGGTGGTCTTCGCCCAGAAGCTGCTGGTGCAGTGGTTTTCAAAGTGGAGTAATGCTGGGGGTGGCCTCAACGGTCGCTGCTAGTCCCGAAGAGGCGAGTGTTACTTCAACAGAAGAAACCTTAACGCCAGCACAGGAGGCCGCACGCACCCGCGCTGCTAACAAAGCCCGAAAGGAAGCTGAGTTGGCTGCTGCCACCGCTGAGCAATAACTAGCATAACCCCTT‑GGGGCCTCTAAACGGGTCTTGAGGGGTTTTTTGCTGAAAGGAGGAACTATATGCGCTCATACGATATGAACGTTGAGACTGCCGCTGAGTTATCAGCTGTGAACGACATTCTGGCGTCTATCGGTGAACCTCCGGTATCAACGCTGGAAGGTGACGCTAACGCAGATGCAGCGAACGCTCGGCGTATTCTCAACAAGATTAACCGACAGATTCAATCTCGTGGATGGACGTTCAACATTGAGGAAGGCATAACGCTACTACCTGATGTTTACTCCAACCTG  >  NC_001604.1/23862‑24458`  

                                                                                                                                                                                                                                                                                                           `|`                                                                                                                                                                                                                                                                                                            
  `aCTGTTAAGCTGCGTGACTTGGCTCTGGAGCGCGCTCGCCGTGCTAACTTCCAAGCGGACCAGATTATCGCTAAGTACGCAATGGGCCACGGTGGTCTTCGCCCAGAAGCTGCTGGTGCAGTGGTTTTCAAAGTGGAGTAATGCTGGGGGTGGCCTCAACGGTCGCTGCTAGTCCCGAAGAGGCGAGTGTTACTTCAACAGAAGAAACCTTAACGCCAGCACAGGAGGCCGCACGCACCCGCGCTGCTAACAAAGCCCGAAAGGAAGCTGAGTTGGCTGCTGCCACCGCTGAGCAATCACt                                                                                                                                                                                                                                                                                                         <  1:257138/301‑1 (MQ=255)`  
            `tGCGTGACTTGGCTCTGGAGCGCGCTCGCCGTGCTAACTTCCAAGCGGACCAGATTATCGCTAAGTACGCAATGGGCCACGGTGGTCTTCGCCCAGAAGCTGCTGGTGCAGTGGTTTTCAAAGTGGAGTAATGCTGGGGGTGGCCTCAACGGTCGCTGCTAGTCCCGAAGAGGCGAGTGTTACTTCAACAGAAGAAACCTTAACGCCAGCACAGGAGGCCGCACGCACCCGCGCTGCTAACAAAGCCCGAAAGGAAGCTGAGTTGGCTGCTGCCACCGCTGAGCAATCACTAGCATAAccc                                                                                                                                                                                                                                                                                               <  1:107848/301‑1 (MQ=255)`  
                       `gCTCTGGAGCGCGCTCGCCGTGCTAACTTCCAAGCGGACCAGATTATCGCTAAGTACGCAATGGGCCACGGTGGTCTTCGCCCAGAAGCTGCTGGTGCAGTGGTTTTCAAAGTGGAGTAATGCTGGGGTTGGCCTCAACGGTCGCTGCTAGTCCCGAAGAGGCGAGTGTTACTTCAACAGAAGAAACCTTAACGCCAGCACATGAGGCCGCACGCACCCGCGCTGCTCACACAGCCCGAAAGGAAGCTGAGTTGGCTGCTGCCACCGCTGAGCAATCACTAGCCTAACCCCTT‑GGGGCctc                                                                                                                                                                                                                                                                                   >  1:215988/1‑301 (MQ=255)`  
                            `tgAGCGCGCTCGCCGTGCTAACTTCCAAGCGGACCAGATTAGTGCTAAGTACGCAATGGGCCACGGTGGTCTTCGCCCAGAAGCTGCTGGTGCAGTGGTTTTCAAAGTGGAGTAATGCTTGGGGTGGCCTCAACGGTCGCTGCTAGTCCCGAAGAGGCGAGTGTTACTTCAACAGAAGAACCCTTAACGCCAGCACAGGAGGCCGCACGCACCCGCGCTGCTAACAAAGCCCGAAAGGAAGCTGAGTTGGCTGCTGCCACCGCTGAGCAATCACTAGCATAACCCCTT‑GGGGCCTCTAAAc                                                                                                                                                                                                                                                                              <  1:244742/300‑1 (MQ=255)`  
                            `ggcgcgcgcTCGACGTGCGAACTTCCAAGCGGACCAGATTATCGCTAAGTACGCAATGGCCCACGGTGTTCTTCGCCCAGAAGCTGCTGGTGCAGTTGTTTTCAAAGTGGAGTAATGCTGGGGGTGGCCTCAACGGTCGCTGCTAGTCCCGAAGAGGCGAGTGTTACTTCAACAGAAGAAACCTTAACGCCAGCACAGGAGGCCGCACGCACCCGCGCTGCTAACAAAGCCCGAAAGGAAGCTGAGTTGGCTGCTGCCACCGCTGAGCAATCACTAGCATAACCCCTT‑GGGGCCTCTAAAc                                                                                                                                                                                                                                                                              <  1:70240/298‑1 (MQ=255)`  
                             `gAGCGCGCTCGCCGTGCTAACTTCCAAGCGGACCAGATTATCGCTAAGTACGCAATGGGCCACGGTGGTCTTCGCCCAGAAGCTGCTGGTGCAGTGGTTTTCAAAGTGGAGTAATGCTGGGGGTGGCCTCAACGGTCGCTGCTAGTCCCGAAGAGGCGAGTGTTACTTCAACAGAAGAAACCTTAACGCCAGCACAGGAGGCCGCACGCACCCGCGCTGCTAACAAAGCCCGAAAGGAAGCTGAGTTGGCTGCTGCCACCGCTGAGCAATCACTAGCATAACCCCTT‑GGGGCCTCTAAACg                                                                                                                                                                                                                                                                             <  1:87755/301‑1 (MQ=255)`  
                               `gcgcgcTCGCCGTGCTAACTTCCAAGCGGACCAGATTATCGCTAAGTACGCAATGGGCCACGGTGGTCTTCGCCCAGAAGCTGCTGGTGCAGTGGTTTTCAAAGTGGAGTAATGCTGGGGGTGGCCTCAACGGTCGCTGCTAGCCCCGAAGAGGCGAGTGTTACTTCAACAGAAGAAACCTTAACGCCAGCACAGGAGGCCGCACGCACCCGCGCTGCTAACAAAGCCCGAAAGGAAGCTGAGTTGGCTGCTGCCACCGCTGAGCAATCACTAGCATAACCCCTT‑GGGGCCTCTAAACggg                                                                                                                                                                                                                                                                           <  1:156887/301‑1 (MQ=255)`  
                               `agagacaggCCGTGCTAACTTCCAAGCGTACCAGATTATCGCTAAGTACGCAATGGGCCACGGTGGTCTTCGCCCAGAAGCTGCTGGTGCAGTGGTTTTCAAAGTGGAGTAATGCTGGGGGTGGCCTCAACGGTCGCTGCTAGTCCCGAAGAGGCGAGTGTTACTTCAACAGAAGAAACCTTAACTCCAGCACAGGAGGCCGCACGCACCCGCGCTGCTAACAAATCCCGAAAGGAAGCTGAGTTGGCTGCTGCCACCGCTGAGCAATCACTAGCATAACCCCTT‑GGGGCCTCTAAACggg                                                                                                                                                                                                                                                                           <  1:191264/293‑1 (MQ=255)`  
                                `cgctcTCGCCGTGCTAACTTCCAAGCGGACCAGATTATCGCTAAGTACGCAATGGGCCACGGTGGTCTTCGCCCAGAAGCTGCTGGTGCAGTGGTTTTCAAAGTGGAGTAATGCTGGGGGTGGCCTAAACGGTCGCTGCTAGTCCCGAAGAGGCGAGTGTTACTTCAACAGAAGAAACCTTAACGCCAGCACAGGAGGCCGCACGCACCTGCGCTGCTAACAAAGCCCGAAAGGAAGCTGAGTTGGCTGCTGCCACCGCTGAGCAATCACTAGCATAACCCCTT‑GGGGCCTCTAAACGGGt                                                                                                                                                                                                                                                                          <  1:317774/301‑1 (MQ=255)`  
                                          `gTGCTAACTTCCAAGCGGACCAGATTATCGCTAAGTACGCAATGGGCCACGGTGGTCTTCGCCCAGAAGCTGCTGGTGCAGTGGTTTTCAAAGTGGAGTAATGCTGGGGGTGGCCTCAACGGTCGCTGCTAGTCCCGAAGAGGCGAGTGTTACTTCAACAGAAGTAACCTTAACGCCAGCACAGGAGGCCGCCCGCACCCGCACTGCTAACCAAGCCCGAAAGGAAGCAGAGTTGGCTGCTGCCACCGCTGAGCACTCTCTAGCATAACCCCTT‑GGGGCCTCTAAACGGGTCTTTAggggc                                                                                                                                                                                                                                                                >  1:31370/1‑300 (MQ=255)`  
                                                 `cTTCCAAGCGGACCAGATTATCGCTAAGTACGCAATGGGCCACGGTGGTCTTCGCCCAGAAGCTGCTGGTGCAGTGGTTTTCAAAGTGGAGTAATGCTGGGGGTGGCCTCAACGGTCGCTGCTAGTCCCGAAGAGGCGAGTGTTACTTCAACAGAAGAAACCTTAACGCCAGCACAGGAGGCCGCACGCACCCGCGCTGCTAACAAAGCCCGAAAGGAAGCTGAGTTGGCTGCTGCAACCGCTGAGCAATCACTAGCATAACCCCTT‑GGGGCCTCTAAAAGGGTCGTGAGGGGGTTTTTGc                                                                                                                                                                                                                                                         >  1:110786/1‑301 (MQ=255)`  
                                                                `gATTATCGCTAAGTACGCAATGGGCCACGGTGGTCTTCGCCCAGAAGCTGCTGGTGCAGTGGTTTTCAAAGTGGAGTAATGCTGGGGGTGGCCTCAACGGTCGCTGCTAGTCCCGAAGAGGCGAGTGTTACTTCAACAGAAGAAACCTTAACGCCAGCACAGGAGGCCGCACGCACCCGCGCTGCTAACAAAGCCCGAAAGGAAGCTGAGTTGGCTGCTGCCACCGCTGAGCAATCACTAGCATAACCCATT‑GGGGCCTCTAATCGGGTCTTGAGGGGTTTTTTGCTGAAAGGAGGAACta                                                                                                                                                                                                                                          >  1:286478/1‑301 (MQ=255)`  
                                                                    `gggagatgtgtataagagacagaCGGTGGTCTTCGCCCAGAAGCTGCTGGTGCAGTGGTTTTCAAAGTGGAGTAATGCTGGGGGTGGCCTCAACGGTCGCTGCTAGTCCCGAAGAGGCGAGTGTTACTTCAACAGAAGAAACCTTAACGCCAGCACAGGAGGCCGCACGCACCCGCGCTGCTAACAAAGCCCGAAAGGAAGCTGAGTTTGCTGCTGCCACCGCTGAGCAATCACTAGCATAACCCCTT‑GGGGCCTCTAAACGGGTCTTGAGGGGTTTTTTGCTGAAAGGAGGAACTATATg                                                                                                                                                                                                                                      <  1:148592/279‑1 (MQ=255)`  
                                                                    `aTCGCTAAGTACGCAATGGGCCACGGTTGTCTTCGCCCAGAAGCTGCTGGTGCAGTGGTTTTCAAAGTGGAGTAATGCTGGGGGTGGCCTCAACGGTCGCTGCTAGTCCCGAAGAGGCGAGTGTTACTTCAACAGAAGAAACCTTAACGCCAGCACAGGAGGCCGCACGCAACCGCGCGGCTAACAAAGCCCGAAAGGAAGCTGAGTTGGCTGCTGCCACCGCTGAGCAATCACTAGCATAACCACTT‑GGGGCCTCTAAACGGGTCTTGAGGGGTTTTTTGATGAAAGGAGGAACtactgg                                                                                                                                                                                                                                      >  1:168566/1‑297 (MQ=255)`  
                                                                        `cTAAGTACGCAATGGGCCACGGTGGTCTTCGCCCAGAAGCTGCTGGTGCAGTGGTTTTCAAAGTGGAGTAATGCTGGGGGTGGCCTCAACGGTCGCTGCTAGTCCCGAAGAGGCGAGTGTTACTTCAACAGAAGAAACCTTAACGCCAGCACAGGAGGCCGCACGCACCCGCGCTGCTAACAAAGCCCGAAAGGAAGCTGAGTTGGCTGCTGCCACCGCTGAGCAATCACTAGCATAACCCCTT‑GGGGCCTCTAAACGGGTCTTGAGTGGTTTTTTGCTGAAAGGAGGAACTATATGCGCt                                                                                                                                                                                                                                  <  1:17895/301‑1 (MQ=255)`  
                                                                        `cTAAGTACGCAATGGGCCACGGTGGTCTTCGCCCAGAAGCTGCTGGTGCAGTGGTTTTCAAAGTGGAGTAATGCTGGGGGTGGCCTCAACGGTCGCTGCTAGTCCCGAAGAGGCGAGTGTTACTTCAACAGAAGAAACCTTAACGCCAGCACAGGAGGCCGCACGCACCCACGCTGCTAACAAAGCCCGAAAGGAAGCTGAGTTGGCTGCTGCCACCGCTGAGCAATCACTAGCATAACCCCTT‑GGGGCCTCTAAACGGGTCTTGAGGGGTTTTTTGCTGAAAGGAGGAACTATATGCGCt                                                                                                                                                                                                                                  <  1:122468/301‑1 (MQ=255)`  
                                                                            `gTACGCAATGGGCCACGGTGGTCTTCGCCCAGAAGCTGCTGGTGCAGTGGTTCTCAAAGTGGAGTAATGCTGGGGGTGGCCTCAACGGTCGCTGCTAGTCCCGAAGAGGCGAGTGTTACTTCAACAGAAGAAACCTTAACGCCAGCACAGGAGGCCGCACGCACCCGCGCTGCTAACAAAGCCCGAAAGGAAGCTGAGTTGGCTGCTGCCACCGCTGAGCAATCACTAGCATAACCCCTT‑TGGGCCTCTAAACGGGTCTTGAGGGGTTTTTTGCTGAAAGGAGGAACTATATGCGCTctgt                                                                                                                                                                                                                              >  1:306346/1‑298 (MQ=255)`  
                                                                               `agacaggggCCACGGTGGTCTTCGCCCAGAAGCTGCTGGTGCAGTGGTTTTCAAAGTGGAGTAATGCTGGGGGTGGCCTCAACGGTCGCTGCTAGTCCCGAAGAGGCGAGTGTTACTTCAACAGAAGAAACCTTAACGCCAGCACAGGAGGCCGCACGCAACCGCGCTGCTAACAAAGCCCGAAAGGAAGCTGAGTTGGCTGCTGCCACCGCTGAGCAATCACTAGCATAACCCCTT‑GGGGCCTCTAAACGGGTCTTGAGGGGTTTTTTGCTGAAAGGAGGAACTATATGCGCTCATGCGa                                                                                                                                                                                                                           <  1:241075/295‑1 (MQ=255)`  
                                                                                 `cAATGTGCCACGGTGGTCTTCGCCCAGAAGCTGCTGGTGCAGTTGTTTTCAAAGTGGAGTAATGCTGGGGGTGGCCTCAACGGTCGCTGCTAGTCCCGAAGAGGCGAGTGTTACTTCAACAGAAGAAACCTTAACGCCAGCACAGGAGGCCGCACGCACCCGCGCTGCTAACAAAGCCCTAAAGGAAGCTGAGTTGGCTGCTGCCACCGCTGAGCAATCACTAGCATAACCCCTT‑GGGGCCTCTAAACGGGTCTTGAGGGGTTTTTTGCTGAAAGGAGGAACTATATGCGCTCATGCGata                                                                                                                                                                                                                         <  1:143156/301‑1 (MQ=255)`  
                                                                                 `cAATGGGCCACGGTGGTCTTCGCCCAGAAGCTGCTGGTGAAGTGGTTTTCAAAGTGGAGTAATGCTGGGGGGGGCCACAACGGTCGCTGCTAGTGCCGAAGAGGCGAGTGTTACTGCAACAGAAGAAACATTAACGCCAGCACAGAAGGCCGCACGCAACCGCGCTGCTAAAAATGCCCGAAAGGAAGCGGAGTTGGATGCTGCCACCGCTGAGCAATCACTAGCATAACGCCTT‑GGGGCCTCTAAACGGGTCTGGAGGGGTTTTTTGCTGATAGGAGGAACTATATgcgcgcgtgtctct                                                                                                                                                                                                                         >  1:315197/1‑291 (MQ=255)`  
                                                                                   `gcaggCCACGGTGGTCTTCGCCAAGAAGCTGCTGGTGCAGTGGTTTTCAAAGTGGAGTAATGCTGGGGGTGGCCTAAACTGTCGCTGCTAGTCCCGAAGAGGCGAGTGTTACTTCAACAGAAGAAACCTTAACGCCAGCACAGGAGGCCGCACGCACCCGCGCTGCTAACAAAGCCCGAAAGGAAGCTGAGTTGGCTGCTGCCACCGCTGAGCAATCACTAGCATAACCCCTT‑GGGGCCTCTAAACGGGTCTTGAGGGGTTTTTTGCTGAAAGGAGGAACTATATGCGCTCATGCGATATg                                                                                                                                                                                                                       <  1:209825/298‑1 (MQ=255)`  
                                                                                   `aTGGGCCACGGTGGTCTTCGCCCAGAAGCTGCTGGTGCAGTGGTTTTCAAAGTGGAGTAATGCTGGGGGTGGCCTCAACGGTCGCTGCTAGTCCCAAAGAGGCTAGTGTTACTTCAACAGAAGAAACCTTAACGACAGCACAGGAGGCCGCACGCACCCGCGCTGCTAACAAAGCACGAAAGGAAGCTGAGTTGGCTGCTGCCACCGCTGAACAATCACTAGCATAACCCATT‑GGGGCCTCTAAACGGGGCTTGAGGGGGTTTTTGCTGATGGGAGGGACTATATGCGCTCATGCGATATg                                                                                                                                                                                                                       >  1:280978/1‑301 (MQ=255)`  
                                                                                     `gggCCACGGTGGTCTTCGCCCAGAAGCTGCTGGTGCAGTGGTTTTCAAAGTGGAGTAATGCTGGGGGTTGCCTCAACGGTCGCTGCTAGTCCCGAAGAGGCGAGTGTTACTTCAACAGAAGAAACCTTAACGCCAGCACAGGAGGCCGCACGCACCCGCGCTGCTAACAAAGCCCGAAAGGAAGCTGAGTTGGCTGCTGCCACCGCTGAGCAATCACTAGCATAACCCCTT‑GGGGCCTCTAAACGGGTCTTGAGGGGTTTTTTGCTGAAAGGAGGAACTATATGCGCTCATGCGATATGaa                                                                                                                                                                                                                     >  1:128240/1‑301 (MQ=255)`  
                                                                                     `gggCCACGGTGGTCTTCGCCCAGAAGCTGCTGGTGCAGTGGTTTTCAAAGTGGAGTAATGCTGGGGGTGGCCTCAACGGTCGCTGCTAGTCCCGAAGAGGCGAGTGTTACTTCAACAGAAGAAACCTTAACGCCAGCACAGGAGGCCGCACGCACCCGCGCTGCTAACAAAGCCCGAAAGGAAGCTGAGTTGGCTGCTGCCACCGCTGAGCAATCACTAGCATAACCCCTT‑GGGGCCTCTAAACGGGTCTTGAGGGGTTTTTTGCTGAAAGGAGGAACTATATGCGCTCATGCGATATGaa                                                                                                                                                                                                                     >  1:125151/1‑301 (MQ=255)`  
                                                                                      `ggCCACGGTGGTCTTCGCCCAGAAGCTGCTGGTGCAGTGGTTTTCAAAGTGGAGTAATGCTGGGGGTGGCCTCAACGGTCGCTGCTAGTCCCGAAGAGGCGAGTGTTACTTCAACAGCAGAAACCTTAACGCCAGCACAGGAGGCCGCACGCACCCGCGCTGCTAACAAAGCCCGAAAGGAAGCTGAGTTGGCTGCTGCCACCGCTGAGCAATCACTAGCATAACCCCTT‑GGGGCCTCTAAACGGGTCTTGAGGGGTTTTTTGCTGAAAGGAGGAACTATATTCGCTCATGCGAAATGAAc                                                                                                                                                                                                                    >  1:230655/1‑301 (MQ=255)`  
                                                                                       `tctaCGGTGGTCTTCGCCCAGAAGCTGCTGGTGCAGTGGTTTTCAAAGTGGAGTAATGCTGGGGGTGGCCTCAACGGTCGCTGCTAGTCCCGAAGAGGCGAGTGTTACTTCAACAGAAGAAACCTTAACGCCAGCACAGGAGGCCGCACGCACCCGCGCTGCTAACAAAGCCCGAAAGGAAGCTGAGTTGGCTGCTGCCACCGCTGAGCAATCACTAGCATAACCCCTT‑GGGGCCTCTAAACGGGTCTTGAGGGGTTTTTTGCTGAAAGGAGGAACTATATGCGCCCATGCGATATGAACg                                                                                                                                                                                                                   >  1:266148/4‑301 (MQ=255)`  
                                                                                          `aCGGTGGTCTTCGCCCAGAAGCTGCTGGTGCAGTTGTTTTCAAAGTGGAGTAATGCTGGGGGTGGCCTCAACTGTCGCTGCTAGTCCCGAAGAGGCGAGTGTTACTTCAACAGAAGAAACCTTAACGCCAGCACAGGAGGCCGCACGCACCCGCGCTGCTAACAAAGCCCGAAAGGAAGCTGAGTTGGCTGCTGCCACCGCTGAGCAATCACTAGCATAACCCCTT‑GGGGCCTCTAAACGGGTCTTGAGGGGTTTTTTGCTGAAAGGAGGAACTATATGCGCTCATGCGATATGAACGttt                                                                                                                                                                                                                >  1:50236/1‑300 (MQ=255)`  
                                                                                              `tggtCTTCGCCCAGGAGCTGATGGTGCAGTGGTTTTCAAAGTGGAGTAATGCTGGGGGTGGCCTCAACGGTCGCTGCTAGTCCCGAAGAGGCGAGCGTTACTTCAACAGAAGAAACCTTAACGCCAGCACAGGAGGCCGCACGCTCCCGCGCTGCTAACAAAGCCCGAAAGGAAGCTGAGTTGGCTGCTGCCACCGCTGAGCAATCACTAGCATAACCCCTT‑GGGGCCTCTAAACGGGTCTTGAGGGGTTTTTTGCTGAAAGGAGGAACTATATGCGCTCATGCGATATGAACGTTGAGAc                                                                                                                                                                                                            <  1:82292/301‑1 (MQ=255)`  
                                                                                              `tggtCTTCGCCCAGAAGCTGCTGGTGCAGTGGTTTTCAAAGTGGAGTAATGCTGGGGGTTGCCTCAACGGTCGCTGCTAGTCCCGAAGAGGCGAGTGTTACTTCAACAGAAGAAACCTTAACGCCAGCACAGGAGGCCGCACGCACCCGCGCTGCTAACAAAGCCCGAAAGGAAGCTGAGTTGGCTGCTGCCACCGCTGAGCAATCACTAGCATAACTCCTT‑GGGGCCTCTAAACGGGTCTTGAGGGGTTTTTTGCTGAAAGGAGGAACTATATGCGCTCATGCGATATGAACGTTGAGAc                                                                                                                                                                                                            <  1:163893/301‑1 (MQ=255)`  
                                                                                                         `cAGAAGCTGCTGGTGCAGTGGTTTTCAAAGTGGAGTAATGCTGGGGGTGGCCTCAACGGTCGCTGCTAGTCCCGAAGAGGCGAGTGTTACTTCAACAGAAGAAACCTTAACGCCAGCACAGGAGGCCGCACGCACCCGCGCTGCTAACAAAGCCCGAAAGGAAGCTGAGTTGGCTGCTGCCACCGCTAAGCAATCACTAGCATAACCCCTT‑GGGGCCTCTAAACGGGTCTTGAGGGGTTTTTTGCTGAAAGGAGGAACTATATGCGCTCATGCGATATGAACGTTTAGACTGCCGCTGAGt                                                                                                                                                                                                 >  1:93598/1‑301 (MQ=255)`  
                                                                                                         `cAGAAGCTGCTGGTGCAGTGGTTTTCAAAGTGGAGTAATGCTGGGGGGGGCCTCAACGGTCGCTGCTAGTCCCGAAGAGGCGAGTGTTACTTCAACAGAAGAAACCTTAACGCCAGCACAGGAGGCCGCACGCACCCGCGCTGCTAACAAAGCCCGAAAGGAAGCTGAGTTGGCTGCTGCCACCGCTGAGCAATCACTAGCATAACCCCTT‑GGGGCCTCTAAACGGGTCTTGAGGGGTTTTTTGCTGAAAGGAGGAACTATATGCGCTCATGCGATATGAACGTTGAGACTGCCGCTGAGt                                                                                                                                                                                                 <  1:274186/301‑1 (MQ=255)`  
                                                                                                           `gAAGCTGCTGGTGCAGTGGTTTTCAAAGTGGAGTAATGCTGGGGGTGGCCTCAACGGTCGCTGCTAGTCCCGAAGAGGCGAGTGTTACTTCAACAGAATAAACCTTAACGCCAGCACAGGAGGCCGCACGCACCTGCGCTGCTAACAAAGCCCGAAAGGAAGCTGAGTTGGCTGCTGCCACCGCTGAGCAATCACTAGCATAACCCATT‑GGGGCCTCTAAACGGGTCTTGAGGGGTTTTTTGCTGAAAGGAGGAACTATATGCGCTCATGCGATATGAACGTTGAGACCGCCCCTGAGTTa                                                                                                                                                                                               >  1:171306/1‑301 (MQ=255)`  
                                                                                                           `aaagaggctgGTGCAGTGGTTTTCAAAGTGGAGTAATGCTGGGGGTGGCCTCAACGGTCGCTGCTAGTCCCGAAGAGGCGAGTGTTACTTCAACAGAAGAAACCTTAACGCCAGCACAGGAGGCCGCACGCACCCGCGCTGCTAACAAAGCCTGAAAGGAAGCTGAGTTGGCTGCTGCCACCGCTGAGCAATCACTAGCATAACCCCTT‑GGGGCCTCTAAACGGGTCTTGAGGGGTTTTTTGCTGAAAGGAGGAACTATATGCGCTCATGCGATATGAACGTTGAGACTGCCGCTGAGTTa                                                                                                                                                                                               <  1:19626/295‑1 (MQ=255)`  
                                                                                                                 `gctgGTGCAGTGGTTTTCAAAGTTGAGTAATGCTGGGGGTGGCCTCAACGGTCGCTGCTAGTCCCGAAGAGGCGAGTGTTACTTCAACAGAAGAAACCTTAACGCCAGCACAGGAGGCCGCACGCACCCGCGCTGCTAACAAAGCCCGAAAGGAAGCTGAGTTGGCTGCTGCCACCGCTGAGCAATCACTAGCATAACCCCTT‑GGGGCCTCTAAACGGGGCTTGAGTGGTTTTTTGCTGAAGGGAGGAACTATCTGCGCTCATGCGATCTGAACTTTGAGACTGCCGCTGAGTTATCAGCt                                                                                                                                                                                         >  1:162982/1‑301 (MQ=255)`  
                                                                                                                 `gctgGTGCAGTGGTTTTCAAAGTGGAGTAATGCTGGGGGTGGCCTCAACGGTCGCTGCTAGTCCCGAAGAGGCGAGTGTTACTTCAACAGAAGAAACCTTAACGCCAGAACAGGAGGCCGCACGCACCCGCGCTGCTAACAAAGCCCGAAAGGAAGCTGAGTTGGCTGCTGCCACCGCTGAGCAATCACTAGCATAACCCCTT‑GGGGCCTCTAAACGGGTCTTGAGGGGTTTTTTGCTGAAAGGAGGAACTATATGCGCTCATGCGATATGAACGTTGAGACTGCCGCTGAgctgtctctt                                                                                                                                                                                         >  1:212689/1‑292 (MQ=255)`  
                                                                                                                            `ggTTTTCAAAGTGGAGTAATGCTTGGGGTGGCCTCAACGGTCGCTGCTAGTCCCGAAGAGGCGAGTGTTACTTCAACAGAAGAAACCTTAACGCCAGCACAGGAGGCAGCACGCACCCGCGCTGCTAACAAAGCCCGAAAGGAAGCTGAGTTGGCTGCTGCCACCGCTGAGCAATCACTAGCATAACCCCTT‑GGGGCCTCTAAACGGGTCTTGAGGGGTTTTTTGCTGAAAGGAGGAACTATATGCGCTCATGCGATATGAACGTTGAGACTGCCGCTGAGTTATCAGCTGTGAACGACAt                                                                                                                                                                              >  1:103650/1‑301 (MQ=255)`  
                                                                                                                            `ggTTTTCAAAGTGGAGTAATGCTGGGGGTGTCCTCAACTGTCGCTGCTAGTCCCGAAGAGGCGAGTGTTACTTCAACAGAAGAAACCTTAACGCCAGCACAGGAGGCCGCACGCACCCGCGCTGCTAACAAAGCCCGAAAGAAAGCTGAGTTGGCTGCTGCCACCGCTGAGCAATCACTAGCATAACCCCTT‑GGGGCCTCTAAACGGGTCTTGAGGGGTTTTTTGCTGAAAGGAGGAACTATATTCGCTCATGCGATCTGAACGTTGAGACTGCCGCTGAGTTATCAGCTGTGACCGACAt                                                                                                                                                                              >  1:184621/1‑301 (MQ=255)`  
                                                                                                                               `tttCAAAGTGGAGTAATGCTGGGGGTGGCCTCAACGGTCGCTGCTAGTCCCGAAGAGGCGAGTGTTACTTCAACAGAAGAAACCTTAACGCCAGCACAGGAGGCCGCACGCACCCGCGCTGCTAACAAAGCCCGAAAGGAAGCTGAGTTGGCTGCTGCCACCGCTGAGCAATCACTAGCATAACCCCTT‑GGGGCCTCTAAACGGGTCTTGAGGGGTTTTTTGCTGAAAGGAGGAACTATATGCGCTCATGCGATATGAACGTTGAGACTGCCGCTGAGTTATCAGCTGTGAACGACATTCt                                                                                                                                                                           >  1:3838/1‑301 (MQ=255)`  
                                                                                                                                 `tCAAAGTGGAGTAATGCTGGGGGTGGCCTCAACGGTCGCTGCTAGTCCCGAAGAGGCGAGTGATACTTCAACAGGAGAAACCTTAACGCCAGCACAGGAGGCCGCACGCACCCGCGCTGCTAACAAAGCCCGAAAGGAAGCTGAGTTGGCTGCTGCCACCGCTGAGCAATCACTAGCATAACCCCTT‑GGGGCCTCTAAACGGGTCTTGAGGGGTTTTTTGCTGAAAGGAGGAACTATATGCGCTCATGCGATATGAACGTTGAGACTGCCGCTGAGTTATCAGCTGTGAACGACATTCTgg                                                                                                                                                                         <  1:3222/301‑1 (MQ=255)`  
                                                                                                                                   `aaaGTGGAGTAATGCTGGGGGTGGCCTCAACGGTCGCTGCTAGTCCCGAAGAGGCGAGTGTTACTTCAACAGAAGAAACCTTAACGCCAGCACAGGAGGCCGCACGCACCCGCGCTGCTAACAAAGCCCGAAAGGAAGCTGAGTTGGCTGCTGCCACCGCTGAGCAATCACTAGCATAACCCCTT‑GGGGCCTCTAAACGGGTCTTGAGGGGTTTTTTGCTGAAAGGAGGAACTATATGCGCTCATGCGATATGAACGTTGAGACTGCCGCTGAGTTATCAGCTGTGAACGACATTATGGCg                                                                                                                                                                       <  1:283986/301‑1 (MQ=255)`  
                                                                                                                                        `ggAGTAATGCTGGGGGTGGCCTCAACGGTCGCTGCTAGTCCCGAAGAGGCGAGTGTTAGTTCAACAGAAGAAACCTTAACGCCAGCACAGGAGGCCGCACGCACCCGCGCTGCTAACAAAGCCCGAAAGGAAGCTGAGTTGGCTGCTGCCACCGCCGAGCAATCACTAGCATAACCCCTT‑GGGGCCTCTAAACGGGTCTTGAGGGGTTTTTTGCTGAAAGGAGGAACTATATGGGCTCATGCGATATGAACGTTGGAACTGCCGATGAGTTATCAGCTGGGAACGAAATTCTGGCGTCCAt                                                                                                                                                                  >  1:206633/1‑301 (MQ=255)`  
                                                                                                                                         `gAGTAATGCTGGGGGTGGCCTCAACGGTCGCTGCTAGTCCCGAAGAGGCGAGTGTTACTTCAACAGAAGAAACCTTAACGCCAGCACAGGAGGCCGCATGCACCCGCGCTGCTAACAAAGCCCGAAAGGAAGCTGAGTTGGCTGCTGCCACCGCTGAGCAATCACTAGCATAACCCCTT‑GGGGCCTCTAAACGGGTCTTGAGGGGTTTTTTGCTGAAAGGAGGAACTATATGCGCTCATGCGATATGAACGTTGAGACTGCCGCTGAGTTATCAGCTGTGAACGACATTCTGGCGTCTATc                                                                                                                                                                 <  1:222857/301‑1 (MQ=255)`  
                                                                                                                                         `gAGTAATGCTGGGGGTGGCCTCAACGGTCGCTGCTAGTCCCGAAGAGGCGAGTGTTACTTCAACAGAAGAAACCTTAACGCCAGCACAGGAGGCCGCACGCACCCGCGCTGCTAACAAAGCCCGAAAGGAAGCTGAGTTGGCTGCTGCCACCGCTGAGCAATCACTAGCATAACCCCTT‑GGGGCCTCTAAACGGGTCTTGAGGGGTTTTTTGCTGCAAGGAGGAACTATATGCGCTCATGCGATATGAACGTTGCGACTGCCGCTGAGTTATCAGCTGTGAACGACATTCTGGCGTCTATc                                                                                                                                                                 >  1:290961/1‑301 (MQ=255)`  
                                                                                                                                         `gAGTAATGCTGGGGGTGGCCTCAACGGTCGCTGCTAGTCCCGAAGAGGCGAGTGTTACTTCAACAGAAGAAACCTTAACGCCAGCACAGGAGGCCGCACGCACCCGCGCTGCTAACAAAGCCCGAAAGGAAGCTGAGTTGGCTGCTGCCACCGCTGAGCAATCACTAGCATAACCCCTT‑GGGGCCTCTAAACGGGTCTTGAGGGGTTTTTTGCTGAAAGGAGGAACTATATGCGCTCATTCGATATGAAAGTTGAGACTGCCGCTGTGTTATCaatgtctcctatacacatctccgagccc                                                                                                                                                                 >  1:331406/1‑274 (MQ=255)`  
                                                                                                                                             `aaTGCTGGGGGTGGCCTCAACGGTCGCTGCTAGTCCCGAAGAGGCGAGTGTTACTTCAACAGAAGAAACCTTAACGCCAGCACAGGAGGCCGCACGCACCCGCGCTGCTAACAAAGCCCGAAAGGAAGCTGAGTTGGCTGCTGCCACCGCTGAGCAATCACTAGCATAACCCCTT‑TGGGCCTCTAAACGGGTCTTGAGGGGTTTTTTGCTGAAAGGAGGAACTATATGCGCTCATGCGATATGAACGTTGAGACTGCCGCTGAGTTATCAGCTGTTAACGACATTCTGGCGTCTATCGGTg                                                                                                                                                             >  1:232995/1‑301 (MQ=255)`  
                                                                                                                                              `aTGCTGGGGGTGGCCTCAACGGTCGCTGCTAGTCCCGAAGAGGCGAGTGTTACTTCAACAGAAGAAACCTTAACGCCAGCACAGGAGGCCGCACGCACCCGCGCTGCTAACAAAGCCCGAAAGGAAGCTGAGTCGGCTGCTGCCACCGCTGAGCAATCACTAGCATAACCCCTT‑GGGGCCTCTAAACGGGTCTTGAGGGGTTTTTTGCTGAAAGTAGGAACTATATGCGCTCATGCGATATGAACGTTGAGACTTCCGCTGAGTTATCAGCTGTGAACGACATTCTGGCGTCTATCGGTGa                                                                                                                                                            >  1:247349/1‑301 (MQ=255)`  
                                                                                                                                                `tcTGTGGGTGGCCTCAACGGTCGCTTCTAGTCCCGAAGAGTCGAGTGTTACTTCAACATAAGAAACCTTCACGCCCGCACTGGAGGCCACACGCACCCGCGCTACTCACGAAGCCCGAAAGTAATCTTCGTTTGCTGCTGCCACCGCTGAGCAATCACTAGCATAACCCCTT‑GTGGCCTCTAAACGGGTCTTGAGGGGTTTTTTGCTGAAATGAGGAACTATATGCGCTCATGCGATATGAACTTTGAGACTGCCGCTGAGTTATCAGCTGTGAACGACATTCTGGCGTCTATCGGTGAAc                                                                                                                                                          <  1:337749/300‑1 (MQ=255)`  
                                                                                                                                                   `gggggTGGCCTCAACGGTCGCTGCTAGTCCCGAAGAGGCGAGTGTTACTTCAACAGAAGAAACCTTAACGCCAGCACAGGAGGCCGCACGCACCCGCGCTGCTAACAAAGCCCGAAAGGAAGCTGAGTTGGCTGCTGCCACCGCTGAGCAATCACTAGCATAACCCCTT‑GTGGCCTCTAAACGGGTCTTGAGGGGTTTTTTGCTGAAAGGAGGAACTATATGCGCTCATGCGATATGAACGTTGAGACTGCCGCTGAGTTATCAGCTGTGAACGACATTCTGGCGTCTATCGGTGAACCTc                                                                                                                                                       >  1:253175/1‑301 (MQ=255)`  
                                                                                                                                                        `gggCCTCAACGGTTGCTGGTAGTCCCGAAGAGGCGAGTGTTACTTCAACAGAAGAAACCTTAACGCCAGAACAGGAGGCCGCACGCACCGGCGCTGCTAACAAAGCCCGAAAGGAAGCTGAGTTGGCTGCTGCCACCGCTGAGCAATCACTAGCAGAACCCCTT‑GGGGCCTCTAAACGGGTCTTGAGGGGTTTTTTGCTGAAAGGAGGAACTATATGCGCTCATGCGATATGAACGTTGAGACTGCCGCTGAGTTATCAGCTGTGAACGACATTCTGGCGTCTATCGGTGAACCTCCGGTa                                                                                                                                                  <  1:324886/300‑1 (MQ=255)`  
                                                                                                                                                         `ggCCTTAACGGTCGCTGCTAGTCCCGAAGAGGCGAGTGTTACTTCAACAGAAGAAACCTTAACGCCAGCACAGGAGGCCGCACGCACCCGCGCTGCTAACAAAGCCCGAAAGGAAGCTGAGTTGGCTGCTGCCACCGCTGAGCAATCACTAGCATAACCCCTT‑GGGGCCTCTAAACGGGTCTTGAGGGGTTTTTTGCTGAAAGGAGGAACTATATGCGCTCATGCGATATGAACGTTGAGACTGCCGCTGAGTTATCAGCTGTGAACGACATTCTGGCGTCTATCGGTGAACCTCCGGTAt                                                                                                                                                 >  1:260787/1‑301 (MQ=255)`  
                                                                                                                                                                    `gcGCTGCTAGTCCCGAAGAGGCGAGTGTTACTTCAACAGAAGAAACCTTAACGCCAGCACAGGAGGCCGCACGCACCCGCGCTGCTAACAAAGCCCGAAAGGAAGCTGAGTTGGCTGCTGCCACCGCTGAGCAATCACTAGCATAACCCCTT‑GGGGCCTCTAAACGGGTCTTGAGGGGTTTTTTGCTGAAAGGAGGAACTATATGCGCTCATGCGATATGAACGTTGAGACTGCCGCTGAGTTATCAGCTGTGAACGACATTCTGGCGTCTATCGGTGAACCTCAGGTATCAACGCTGGaa                                                                                                                                      <  1:52001/300‑1 (MQ=255)`  
                                                                                                                                                                     `cGCTGCTAGTCCCGAAGAGGCGAGTGTTACTTCAACAGAAGAAACCTTAACGCCAGCACAGGAGGCCGCACGCACCCGCGCTGCTAACAAAGCCCGAAAGGAAGCTGAGTTGGCTGCTGCCACCGCTGAGCAATCACTAGCATAACCCCTT‑GGGGCCTCTAAACGGGGCTTGAGGGGTTTTTTGCTGAAAGGAGGAACTGTATGCGCTCATGCGATATGAACGTTGAGACTGCCGCTGAGTTATCAGCTGTGAACGACATTCTGGCGTCTATCGGTGAACCTCCGGTATAAACGCTGGAAg                                                                                                                                     >  1:75487/1‑301 (MQ=255)`  
                                                                                                                                                                       `ctgctAGTCCCGAAGAGGCGAGTGTTACTTCAACAGAAGAAACCTTAACGCCAGCACAGGAGGCCGCACGCACCCGCGCTGCTAACAAAGCCCGAAAGGAAGCTGAGTTGGCTGCTGCCACCGCTTAGCAATCACTAACATAACCCCTT‑GGGGCCTCTAAACGGGTCTTGAGTTGTTTTTTGCTGAAAGGCGGAACTATATGCGCTCCTGCGATATGAACGTTGAGACTGCCGCTGAGTTATCAGCTGTGAACGTCATTCTGGCGTCTATCGTTTAACCTCCGGTATCAACGCTTGAAggg                                                                                                                                   >  1:145496/1‑300 (MQ=255)`  
                                                                                                                                                                           `aaGTCCCGGAGAAGCGAGTGATACGGCAAAAGAAGAAGCCTTAACGCCAGCACAGGAGGCCGCACGCACCCGCGCTGGTAACAAAGCCCGAAGGGAAGCTGAGTTGGCTGCTGCCAGCGCTGAGCAATGACTAGCATAACCGCTT‑GGGGCCTCTAAGCGGGTCTTGAGGGGTTTTTTGCTGAAAGGAGGAACTATATGCGCTCATGCGATATGAACGTTGAGACTGCCGCTGAGTTATCAGCTGTGAACGACATTCTGGCGTCTATCGGTGAACCTCCGGTATCAACGCTGGAAGGTGACg                                                                                                                               <  1:304068/300‑1 (MQ=255)`  
                                                                                                                                                                                  `aaaGAGGCGAGTGTTACTTCAACAGAAGAAACCTTAACGCCAGCACAGGAGGCCGCACGCCCCCGCGCTGCTAACAAAGCCCGAAAGGAAGCTGAGTTGGCTGCTGCCACCGCGGAGCAATCACTAGCATAACCCCTT‑GGGGCCTCTAAACGGGTCTTGAGGGGTTTTTTGCTGAAAGGAGGAACTATATGCGCTCATGCGATATGAACGTTGAGACTGCCGCTGAGTTATCAGCTGTGAACGACATTCTGGCGTCTATCGGTGAACCTCCGGTATCAACGCTGGAAGGTGACGCTAACGc                                                                                                                        <  1:4823/300‑1 (MQ=255)`  
                                                                                                                                                                                   `aaGAGGCGAATGTTACTTAAACAGAAGAAACCTTAACGCCAGCACAGGAGGCCGCACGCACCCGCGCTGCTAACAAAGCCCGAAAGGAAGCTGAGTTGGCTGCTGCCACCGCTGAGCAATCACTAGCATAACCCCTT‑‑GGGCCTCTAAACGGGTCTTGAGTGGTTTTTTGCTGAAAGGAGGAACTATATGCGCTCATGCGATATGAACGTTGAGACTGCCGCTGAGTTATCAGCTGTGAACGACATTCTGGCGTCTATCGGTGAACCTCCGGTATCAACGCTGGAAGGTGACGCTAACGCAg                                                                                                                      <  1:44753/301‑1 (MQ=255)`  
                                                                                                                                                                                    `agagGCGAGTGTTACTTCAACAGAAGAAACCTTAACGCCAGCACAGGAGGCCGCACGCACCCGCGCTGCTAACAAAGCCCGAAAGGAAGCTGAGTTGGCTGCTGCCACCGCTGAGCAATCACTAGCATAACCCCTT‑GGGGCCTCTAAACGGGTCTTGAGTGGTTTTTTGCTGAAAGGAGGAACTATATGCGCTCATGCGATATGAACGTTGAGACTGCCGCTGAGTTATCAGCTGTGAACGACATTCTGGCGTCTATCGGTGAACCTCCGGTATCAACGCTGGAAGGTGACGCTAACGCAg                                                                                                                      <  1:23682/301‑1 (MQ=255)`  
                                                                                                                                                                                        `gCGAGTGTTACTTCAACAGAAGAAACCTTAACGCCAGCACAGGAGGCCGCACGCACCCGCGCTGCTAACAAAGCCCGAAAGGAAGCTGAGTTGGCTGCTGCCACCGCTGAGCAATCACTAGCATAACCCCTT‑GGGGCCTCTAAACGGGTCGTGAGGGGTTTTGTGCGGAAAGGAGGAACGATATGCGCTCATGCGATATGAACGTTGAGACTGCCGCTGAGTTATCAGCTGTGAACGACATTCTGGCGTCTATCGGTGAACCTCCGGTATCAACGCTGGAAGGTGACGCTAACGCAGATGc                                                                                                                  <  1:119646/301‑1 (MQ=255)`  
                                                                                                                                                                                            `gtgtTACTTCAACAGAAGAAACCTTAACGCCAGCACAGGAGGCCGCACGCACCCGCGCTGCTAACAAAGCCCGAAAGGAAGCTGAGTTGGCTGCTGCCACCGCTGAGCAATCACTAGCATAACCCCTT‑GGGGCCTCTAAACGGGTCTTGAGGGGTTTTTTGCTGAAAGGAGGAACTATATGCGCTCATGCGATATGAACGTTGAGACTGCCGCTGAGTTATCAGCTGTGAACGACATTCTGGCGTCTATCGGTGAACCTCCGGTATCAACGCTGGAAGGTGACGCTAACGCAGATGCAGCg                                                                                                              >  1:1005/1‑301 (MQ=255)`  
                                                                                                                                                                                                       `aCAGAAGAAACCTTAACGCCAGCACAGGAGGCCTCACGCACCCGCGCTGCTAACAAAGCCCGAAATGAAGCTGAGTTGGCTGCTGCCACCGCTGAGCAATCACTAGCATAACCCCTT‑GGGGCCTCTAAACGGGTCTTGAGGGGTTTTTTGCTGAAAGGAGGAACTATATGCGCTCATGCGATATGAACGTTGAGACTGCCGCTGAGTTATCAGCTGTGAACGACATTCTGGCGTCTATCGGTGAACCTCCGGTATCAACGCTGGAAGGTGACGCTAACGCAGATGCAGCGAACGCTCGGCg                                                                                                   <  1:193769/301‑1 (MQ=255)`  
                                                                                                                                                                                                          `gaagaaACCTTAACGCCAGCACAGGAGGCCGCACGCACCCGCGCTGCTAACAAAGCCCGAAAGGAAGCTGAGTTGGCTGCTGCCACCGCTGAGCAATCACTAGCATAACCCCTT‑GGGGCCTCTAAACGGGTCTTGAGAGGTTTTTTGCTGAAAGGAGGAACTATATGCGCTCATGCGATATGAACGTTGAGACTGCCGCTGAGTTATCAGCTGTGAACGACATTCTGGCGTCTATCGGTGAACCTCCGGTATCAACGCTGGAAGGTGACGCTAACGCAGATGCAGCGAACGCTCGGCGTAt                                                                                                >  1:141088/1‑301 (MQ=255)`  
                                                                                                                                                                                                          `gaagaaACCTTAACGCCAGCACAGGAGGCCGCACGCACCCGCGCTGCTAACAAAGCCCGAAACGAAGCTGAGTTGGCTGCTGCCACCGCTGAGCAATCACTAGCATAACCCCTT‑GGGGCCTCTAAACTGGTCTTGAGGGGTTTTTTGCTGAAAGGAGGAACTATATGCGCTCATGCGATATGAACGTTGAGACTGCCGCTGAGTTATCAGCTGTGAACGACATTCTGGCGTCTATCGGTGAACCTCCGGTATCAACGCTTGAAGGTGACGCTAACGCGGATGCAGCGAACGCCCGTCGTAt                                                                                                >  1:152127/1‑301 (MQ=255)`  
                                                                                                                                                                                                                `aCCTTAACGCCAGCACAGGAGGCCGCACGCACCCGCGCTGCTAACAAAGCCCGAAAGGAAGCTGAGTAGGCTGCTGCCACCGCTGAGCAATCACTAGCATAACCCCTT‑GGGGCCTCTAAACGGGTCTTGAGGGGTGTTTTGCTGAAAGGAGGAACTATATGCGCTCATGCGATATGAACGTTGAGACTGCCGCTGAGTTATAAGCTGTTAACGACATTCTGGCGTCTATCGGGTAACCTCCGGTATCAACGCTGGAAGGTGACGCTAACGCAGAGGCAGCGAACGCTCGGCGTATTCTcaa                                                                                          >  1:188211/1‑301 (MQ=255)`  
                                                                                                                                                                                                                 `ccTTAACGCCAGCACAGGAGGCCGCACGCACCCGCGCTGCTAACAAAGCCCGAAAGGAAGCTGAGTTGGCTGCTGCCACCGCTGAGCAATCACTAGCATAACCCCTT‑GGGGCCTCTAAACGGGTCTTGAGGGGTTTTTTGCTGAAAGGAGGAACTATATGCGCTCATGCGATATGAACGTTGAGACTGCCGCTGAGTTATCAGCTGTGAACGACATTCTGGCGTCTATCGGTGAACCTCCGGTATCAACGCTGGAAGGTGACGCTAACGCAGATGCAGCGAACGCTCGGCGTATTCTcacc                                                                                         <  1:66610/301‑3 (MQ=255)`  
                                                                                                                                                                                                                 `ccTTAACGCCAGCACAGGAGGCCGCACGCACCCGCGCTGCTAACAAAGCCCGAAAGGAAGCTGAGTTGGCTGCTGCCACCGCTGAGCAATCACTAGCATAACCCCTT‑GGGGCCTCTAAACGGGTCTTGAGGGGTTTTTTGCTGAAAGGAGGAACTATATGCGCTCATGCGATATGAACGTTGAGACTGCCGCTGAGTTATCAGCTGTGAACGACATTCTGGCGTCTATCGGTGAACCTCAGGTATCAACGCTGGAAGGTGACGCTAACGCAGATGCAGCGAACGCTCGGCGTATTCTcaac                                                                                         <  1:79147/301‑1 (MQ=255)`  
                                                                                                                                                                                                                  `cTTAACGCCAGCACAGGAGGCCGCACGCACCCGCGCTGCTAACAAAGCCAGAAAGGAAGCTGAGTTGGCTGCTGCCACCGCTGAGCAATCACTAGCATAACCCCTT‑GGGGCCTCTAAACGGGTATTGAGGGGTTTTTTGCTGAAAGGAGGAACTATATGCGCTCATGCGATATGAACGTTGAGACTGCAGCTGAGTTATCAGCTGTGAACGACATTCTGGCGTCTATCGGTGAACCTCCGGTATCAACGCTGGAAGGTGACGCTAACGCAGATGCAGCGAACGCTCGGCGTATTCTcaaca                                                                                        >  1:292527/1‑301 (MQ=255)`  
                                                                                                                                                                                                                  `aataagagacagacaGGAGGCCGCACGCACCCGCGCTGCTAACAAAGCCCGAAAGGAAGCTGAGTTGGCTGCTGCCACCGCTGAGCAATCACTAGCATAACCCCTTGGGGGCCTCTAAACGGGTCTTGAGGGGTTTTTTGCTGAAAGGAGGAACTATATGCGCTCATGCGATATGAACGTTGAGACTGCCGCTGAGTTATCAGCTGTGAACGACATTCTGGCGTCTATCGGTGAACCTCCGGTATCAACGCTGGAAGGTGACGCTAACGCAGATGCAGCGAACGCTCGGCGTATTCTcaac                                                                                         <  1:41781/289‑1 (MQ=255)`  
                                                                                                                                                                                                                      `aCGCCAGCACAGGAGGCCGCACGCACCCGCGCTGCTAACAAAGCCCGAAAGGAAGCTGAGTTGGCTGCTGCCACCGCTGAGCAATCACTAGCATAACCCATT‑GGGGCCTCTAAACGGGTCTTGAGGGGTTTTTTGCTGAAAGGAGGAACTATATGCGCTCTTGCGATATGAACGTTGAGACTGCCGCTGAGTTATCAGCTGTGAACGACATTCTGGCGTCTATCGGTGAACCTCCGGTATCAACGCTGGAAGGTGACGCTAACGCAGATGCAGCGAACGCTCGGCGTATTCTCAACAAGAt                                                                                    <  1:295695/301‑1 (MQ=255)`  
                                                                                                                                                                                                                          `cAGCACAGGAGGCCGCACGCACCCGCGCTGCTAACAAAGCCCGAAAGGAAGCTGAGTTGGCTGCTGCCACCGCTGAGCAATCACTAGCATAACCCCTT‑GGGGCCTCTAAACGGGTCTTGAGGGGTTTTTTGCTGAAAGGAGGAACTATATTCGCTCATGCGATATGAACGTTGAGACTGCTGCTGAGTTATCAGCTGTGAACGACATTCTGGCGTCTATCGGTGAACCTCCGGTATCAACGCTGGAAGGTGACGCTAACGCAGATGCAGCGAACGCTCGGCGTATTCTCAACAAGATTAAc                                                                                <  1:7291/301‑1 (MQ=255)`  
                                                                                                                                                                                                                            `gCACAGGCGTCCGCCCTCCCCCGCGCTGCTAACAAAGCCCGAAAGGAAGCTGAGTTGGCTGCTGCCACCGCTGAGCAATCACTAGCATAACCCCTT‑GGGGCCTCTAAACGGGTCTTGAGGGGTTTTTTGCTGAAAGGAGGAACTATATGCGCTCATGCGATATGAACGTTGAGACTGCCGCTGAGTTATCAGCTGTGAACGACATTCTGGCGTCTATCGGTGAACCTCCGGTATCAACGCTGGAAGGTGACGCTAACGCAGATGCAGCGAACGCTCGGCGTATTCTCAACAAGATTAACCg                                                                              >  1:335164/1‑301 (MQ=255)`  
                                                                                                                                                                                                                            `gCACAGGAGGCCGCATGCACCCGCGCTGCTAACAAAGCCCGAAAGGAAGCTGAGTTGGCTGCTGCCACCGCTGAGCAATCACTAGCATAACCCCTT‑GGGGCCTCTAAACGGGTCTTGAGGGGTTTTTTGCTGAAAGGAGGAACTATATGCGCTCATGCGATATGAACGTTGAGACTGCCGCTGAGTTATCAGCTGTGAACGACATTCTGGCGTCTATCGGGGAACCTCCGGTATCAACGCTGGAAGGTGACGCTAACGCAGATGCAGCGAACGCTCGGCGTATTCTCAACAAGATTAAcag                                                                              >  1:166969/1‑299 (MQ=255)`  
                                                                                                                                                                                                                            `gCACAGGAGGCCGCACGCACCCGCGCTGCTAACAAAGCCCGAAAGGAAGCTGAGTTGGCTGCTGCCACCGCTGAGCAATCACTAGCATAACCCCTT‑GGGGCCTCTAAACGGGTCTTGAGGGGTTTTTTGCTGAAAGGAGGAACTATATGCGCTCATGCGATATGAACGTTGAGACTGCCGCTGAGTTATCAGCTGTGAACGACATTCTGGCGTCTATCGGTGAACCTCCGGTATCAACGCTGGAAGGTGACGCTAACGCAGATGCAGCGAACGCTcctgtctcttatacacatctccgagc                                                                              >  1:113415/1‑276 (MQ=255)`  
                                                                                                                                                                                                                            `gCACAGGAGGCCGCACGCACCCGCGCTGCTAACAAAGCCCGAAAGGAAGCTGAGTTGGCTGCTGCCACCGCTGAGCAATCACTAGCATAACCCCTT‑GGGGCCTCTAAACGGGTCTTGAGGGGTTTTTTGCTGAAAGGAGGAACTATATGCGCTCATGCGATATGAACGTTGAGACTGCCGCTGAGTTATCAGCTGTGAACGACATTCTGGCGTCTATCGGTGAACCTCCGGTATCAACGCTGGAAGGTGACGCTAACGCAGATGCAGCGAACGCTCGGCGTATTCCCAACAAGATTAACCg                                                                              >  1:278104/1‑301 (MQ=255)`  
                                                                                                                                                                                                                               `caGGAGGCCGCACGCACCCGCGCTGCTAACAAAGCCCGAAAGGAAGCTGAGTTGGCTGCTGCCACCGCTGAGCAATCACTAGCATAACCCCTT‑GGGGCCTCTAAACGGGTCTTGAGGGGTTTTTTGCTGAAAGGAGGAACTATATGCGCTCATGCGATATGAACGTTGAGACTGCCGCTGAGTTATCAGCTGTGAACGACATTCTGGCGTCTATCGGTGAACCTCCGGTATCAACGCTGGAAGGTGACGCTAACGCAGATGCAGCGAACGCTCGGCGTATTCTCAACAAGAtctgtctctt                                                                           >  1:263254/1‑292 (MQ=255)`  
                                                                                                                                                                                                                                      `ccGCACGCACCCGCGCTGCTAACAAAGCCCGAAAGGAAGCTGAGTTGGCTGCTGCCACCGCTGAGCAATCACTAGCATAACCCCTT‑GGGGCCTCTAAACGGGTCTTGAGGGGTTTTTTGCTGAAAGGAGGAACTATATGCGCTCATGCGATATGAACGTTGAGACTGCCGCTGAGTTATCAGCTGTGAACGACATTCTGGCGTCTATCGGTGAACCTCCGGTATCAACGCTGGAAGGTGACGCTAACGCAGATGCAGCGAACGCTCGGCGTACTCTCAACAAGAtctggctcttctacaca                                                                    >  1:59361/1‑285 (MQ=255)`  
                                                                                                                                                                                                                                      `ccGCACGCACCCGCGCTGCTAACAAAGCCCGAAAGGAAGCTGAGTTGGCTGCTGCCACCGCTGAGCAATCACTAGCATAACCCCTT‑GGGGCCTCTAAACGGGTCTTGAGGGGTTTTTTGCTGAAAGGAGGAACTATATGCGCTCATGCGATATGAACGTTGAGACTGCCGATGAGTTATCAGCTGTGAACGACATTCTGGCGTCTATCGGTGAACCTCCGGTATCAACGCTGGAAGGTGACGCTAACGCAGATGCAGCGAACGCTCGGCGTATTCTCAACAAGATTAACCGACAGATTCaa                                                                    >  1:249969/1‑301 (MQ=255)`  
                                                                                                                                                                                                                                        `gcatgcacCCGCGCTGCTAACAAAGCCCGAAAGGAAGCTGAGTTGGCTGCTGCCACCGCTGAGCAATCACTAGCATAACCCCTT‑GGGGCCTCTAAACGGGTCTTGAGGGGTTTTTTGCTGAAAGGAGGAACTATATGCGCTCATGCGATATGAACGTTGAGACTGCCGCTGAGTTATCAGCTGTGAACGACATTCTGGCGTCTATCGGTGAACCTCCGGTATCAACGCTGGAAGGTGACGCTAACGCAGATGCAGCGAACGCTCGGCGTATTCTCAACAAGATTAACCGACAGATTCAAtc                                                                  >  1:17481/1‑301 (MQ=255)`  
                                                                                                                                                                                                                                        `gcacgcacCCGCGCTGCTAACAAAGCCCGAAAGGAAGCTGAGTTGGCTGCTGCCACCGCTGAGCAATCACTAGCATAACCCCTT‑GGGGCCTCTAAACGGGTCTTGATGGGTTTTTTGCTGAAAGGAGGAACTATATGCGCTCATGCGATATGAACGTTGAGACTGCCGCTGAGTTATCAGCTGTGAACGACATTCTGGCGTCTATCGGTGAACCTCCGGTATCAACGCTGGAAGGTGACGCTAACGCAGATGCAGCGAACGCTAGGCGTATTCTCAACAAGATTAACCGACAGATTCAAtc                                                                  <  1:288508/301‑1 (MQ=255)`  
                                                                                                                                                                                                                                             `cacCCGCGCTGCTAACAAAGCCCGAAAGGAAGCTGAGTTGGCTGCTGCCACCGCTGAGCAATCACTAGCATAACCCCTT‑GTGGCCTCTAAACGGGTCTTGAGGGGTTTTTTGCTGAAAGGAGGAACTATATGCGCTCATGCGATATGAACGTTGAGACTGCCGCTGAGTTATCAGCTGTGAACGACATTCTTGCGTCTATCGGTGAACCTCCGGTATCAACGCTGGAGGGTGACGCTAACGCAAATGCAGCGAACGCTCGGCGTATTCTCAAAAAGATTAACCGACAGATTCAATATCGtg                                                             >  1:136344/1‑301 (MQ=255)`  
                                                                                                                                                                                                                                             `cacCCGCGCTGCTAACAAAGCCCGAAAGGAAGCTGAGTTGGCTGCTGCCACCGCTGAGCAATCACTAGCATAACCCCTT‑GTGGCCTCTAAACGGGTCTTGAGGGGTTTTTTGCTGAAAGGAGGAACTATATGCGCTCATGCGATATGAACGTTGAGACTGCCGCTGAGTTATCAGCTGTGAACGACATTCTGGCGTCTATCGGTGAACCTCCGGTATCAACGCTGGAAGGTGACGCTAACGCAGATGCAGCGAACGCTCGGCGTATTCTCAACAAGATTAACCGACAGATTCAATCTCGtg                                                             >  1:270181/1‑301 (MQ=255)`  
                                                                                                                                                                                                                                             `cacCCGCGCTGCTAACAAAGCCCGAAAGGAAGCTGAGTTGGCTGCTGCCACCGCTGAGCAATCACTAGCATAACCCCTT‑GGGGCCTCTAAACGGGTCTTGAGGGGTTTTTTGCTGAAAGGAGGAACTATATGCGCTCATGCGATATGAACGTTGAGACTGCCGCTGAGTTATCAGCTGTGAACGACATTCTGGCGTCTATCGGTGAACCTCCGGTATCAACGCTGGAAGGTGACGCTAACGCAGATGCAGCGAACGCTCGGCGTATTCTCAACAAGATTAACCGACAGATTCAATCCCGtg                                                             >  1:22667/1‑301 (MQ=255)`  
                                                                                                                                                                                                                                                       `gctAACAAAGCCCGAAAGGAAGCTGAGTTGGCTGCTGCCACCGCTGAGCAATCACTAGCATAACCCCTT‑GGGGCCTCTAAACGGGTCTTGAGGGGCTCTTTGCTCAAAGGAGGAACTATATGCGCTCATGCGATATGAACGTTGAGACCGCCGCTGAGTTATCAGATGTGAACGACATTCTGGCGTCTATCGGTGAACCTCCGGTATCAACGCTGGAAGGTGACGCTAACGAAGATTCAGAGACCGCTCGGCGCATTCCCAACAAGATTAACCGACAGATTCAATATCGTGGATGGCCGtt                                                   >  1:199447/1‑301 (MQ=255)`  
                                                                                                                                                                                                                                                               `aGCCTGAAAGGAAGCTGAGTTGGCTGCTGCCACCGCTGAGCAATCACTAGCATAACCCCTT‑GGGGCCTCTAAACGGGTCTTGAGGGGTTTTTTGCTGAAAGGAGGAACTATATGCGCTCATGCGATATGAACGTTGAGACTGCCGCTGAGTTATCAGCTGTGAACGACATTCTGGCGTCTATCGGTGAACCTCCGGTATCAACGCTGGAAGGTGACGCTAACGCAGATGCAGCGAACGCTCGGCGGATTCTCAACAAGATTAACCGACAGGTTCAATCTCGtggatggcatgtcgcttata                                           >  1:299121/1‑288 (MQ=255)`  
                                                                                                                                                                                                                                                                `gCCCGAAAGGAAGCTGAGTTGGCTGCTGCCACCGCTGAGCAATCACTAGCATAACCCCTT‑GGGGCCTCTAAACGGGTCTTGAGGGGTTTTTTGCTGAAAGGAGGAACTATATGCGCTCATGCGATATGAACGTTGAGACTGCCGCTGAGTTATCAGCTGTGAACGACATTCTGGCGTCTATCGGTGAACCTCCGGTATCAACGCTGGAAGGTGAAGCTAAAGCAGATGTAGCGAACGCACGGCGTATTCTCAACAAGATAAACCGACAGATTCAATCTCGCGGATGGACGTTCAACATTGa                                          >  1:301851/1‑301 (MQ=255)`  
                                                                                                                                                                                                                                                                `gCCCGAAAGGAAGCTGAGTTGGCTGCTGCCACCGCTGAGCAATCACTAGCATAACCCCTT‑GGGGCCTCTAAACGGGTCTTAAGGGGTTTTTTGCTGAAAGGAGGAACTATATGCGCTCATGCGATATGAACGTTGAGACTGCCGCTGAGTTATCAGCTGTGAACGACATTCTGGCGTCTATCGGTGAACCTCCGGTATCAACGCTGGAAGGTGACGCTAACGCAGATGCAGCGAACGCTCGGCGTATTCTCAACAAGATTAACCGACAGATTCAATCTCGTGGATGGACGTTCAACATTGa                                          >  1:133360/1‑301 (MQ=255)`  
                                                                                                                                                                                                                                                                  `ccGAAAGGAAGCTGAGTTGGCTGCTGCAACCGCTGAGCAATCACTAGCATAACCCCTG‑GGGGCCTCTAAACGGGTCTTGAGGGGTTTTTTGCTGAAAGGAGGAACTATATGCGCTCATGCGATATGAACTTTGAGACTGCCGCTGAGTTATCAGCTGTGAACGACATTCTGGCGTCTATCGGTGAACCTCCGGTATCAACGCTGGAAGGTGACGCTAACGCAGATGCAGCGAACGCTCGGCGTATTCTCAACAAGATTAACCGACAGATTCAATCTCGTGGATGGACGTTCAACATTGAgg                                        <  1:329102/301‑1 (MQ=255)`  
                                                                                                                                                                                                                                                                               `gAGTTGGCTGCTGCCACCGCTGAGCAATCACTAGCATAACCCCTT‑GGGGCCTCTAAACGGGTCTTGAGGGGTGTTTTGCTGAAAGGAGGAACTATATGAGCCCATGCGATATTAACGGTGAGACTGCCGCTGAGTTATCAGCTGTGAACGACATTCGGGAGTCTATCGGTGAACCTCCGGTAGAAACGCTGGAAGGTGACGCTAACCCAAATGCAGCCACCGCTCGGCGTATTCTCAACACGATTAACCGACAGATTCAAACTCGGCGAGGGACATTCACAATTGAGGGAGGGCTAACGca                           >  1:18197/1‑300 (MQ=255)`  
                                                                                                                                                                                                                                                                                   `tggctacgtccACCGCTGAGCAATTACTAGCATAACCCCTT‑GGGGCCTCTAAACGGGTCTTGAGGGGTTTTTTGCTGAAAGGAGGAACTATATGCGCTCATGCGATATGAACGTTGAGACTGCCGCTGAGTTATCAGCTGTGAACGACATTCTGGCGTCTATCGGTGAACCTCCGGTATCAACGCTGGAAGGTGACGCTAACGCAGATGCAGTGAACGCTCGGCGTATTCTCAACAAGATTAACCGACAGATTCAATCTCGTGGATGGACGTTCAACATTGAGGAAGGCATAACGctacta                       <  1:203089/292‑1 (MQ=255)`  
                                                                                                                                                                                                                                                                                    `ggCTGCTGCGACCGCTGAGCAATCACTAGCATAACCCCTT‑GGGGCCTCTAAACGGGTCTTGAGGGGTTTTTTGCTGAAAGGAGGAACTATATGCGCTCATGCGATATGAACGTTGAGACTGCCGCTGAGTTATCAGCTGTGAACGACATTCTGGCGTCTATAGGTGAACCTCCTGTATCAACGCTGGAAGGTGACGCTAACACAGATGCAGCGAACGCTCGGCGTATTCTCAACAAGATTAACCGACAGATTCAATCTCGTGGATGGACGTTCAACATTGAGGAAGGCATAACGCtactac                      <  1:54429/301‑1 (MQ=255)`  
                                                                                                                                                                                                                                                                                     `gctgctGCCACCGCTGAGCAATCACTAGCATAACCCCTT‑GGGGCCTCTAAACGGGTCTTGAGGGGTTTTTTGCTGAAAGGAGGAACTATATGCGCTCATGCGATATGAACGTTGAGACTGCCGCTGAGTTATCAGCTGTGAACGACATTCTGGCGTCTATCTGTGAACCTCCGGTATCAACGCTGGAAGGTGACGCTAACGCAGATGCAGCGAACGCTCGGCGTATTCTCAACAAGATTAACCGACAGATTCAATCTCGTGGATGGACGTTCAACATTGAGGAAGGCATAACACTACTAcc                     <  1:86351/301‑1 (MQ=255)`  
                                                                                                                                                                                                                                                                                      `ctgctgCCACCGCTGAGCAATCACTAGCATAACCCCTT‑GGGGCCTCTAAACGGGTCTTGAGGGGTTTTTTGATGAAAGGAGGAACTATATGCGCTCATGCGATATGAACGTTGAGACTGCCGCTGAGTTATCAGCTGTGAACGACATTCTGGCGTCTATCGGTGAACCTCCGGTATCAACGCTGGAAGGTGACGCTAACGCAGATGCAGCGAACGCTCGGCGTATTCTCAACAAGATTAACCGACAGATTCAATCTCGTGGATGGACGTTCAACATTGAGGAAGGCATAACGCTACTACCt                    <  1:90433/301‑1 (MQ=255)`  
                                                                                                                                                                                                                                                                                      `ctgctgCAACCGCTGAGCAATCACTAGCATAACCCCTT‑GGGGCCTCTAAACGGGTCTTGAGGGGTTTTTTGCTGAAAGGAGGAACTATATGCGCTCATGCGATATGAACGTTGAGACTGCCGCTGAGTTATCAGCTGTGAACGACATTCTGGCGTCTATCGGTGAACCTCCGGTATCAACGCTGGAAGGTGACGCTAACGCAGATGCAGCGAACGCTCGGCGTATTCTCAACAAGATTAACCGACAGATTCAATCTCGTGGATGGACGTTCAACATTGAGGAAGGCATAACGCTACTACCt                    <  1:15985/301‑1 (MQ=255)`  
                                                                                                                                                                                                                                                                                      `aagagacagccGCTGAGCAATCACTAGCATAACCCCTT‑GGGGCCTCTAAACGGGTCTTGAGGGGTTTTTTGCTGAAAGGAGGAACTATATGCGCTCATGCGATATGAACGTTGAGACTGCCGCTGAGTTATCAGCTGTGAACGACATTCTGGCGTCTATCGGTGAACCTCCGGTATCAACGCTGGAAGGTGACGCTAACGCAGATGCAGCGAACGCTCGGCGTATTCTCAACAAGATTAACCGACAGATTCAATCTCGTGGATGGACGTTCAACATTGAGGAAGGCATAACGCTACTACCt                    <  1:115718/292‑1 (MQ=255)`  
                                                                                                                                                                                                                                                                                         `ctgcCACCGCTGAGCAATCACTAGCATAACCCCTT‑GGGGCCTCTAAACGGGTCTTGAGGGGTTTTTTGCTGAAAGGAGGAACTATATGCGCTCATGCGATATGAACGTTGAGACTGCCGCTGAGTTATCAGCTGTGAACGACATTCTGGCGTCTATCGGTGAACCTCCGGTATCAAAGCTGGAAGGTGACGCTAACGCAGATGCAGCGAACGCTCGGCGTATTCTCAACAAGATTAACCGACAGATTCACTCTCGTGGATGGACGTTCAACATTAAGGAAGGCATAACGCTACTACCTGAt                 >  1:96966/1‑301 (MQ=255)`  
                                                                                                                                                                                                                                                                                         `ctgcCACCGCTGAGCAATCACTAGCATAACCCCTT‑GGGGCCTCTAAACGGGTCTTGAGGGGTTTTTTGCTGAAAGGAGGAACTATATGCGCTCATGCGATATGAACGTTGAGACTGCCGCTGAGTTATCAGCTGTGAACGACATTCTGGCGTCTATCGGTGAACCTCCAGTATCAACGCTGGAAGGTGACGCTAACGCAGATGCAGCGAACGCTCGGCGTATTCTCAACAAGATTAACCGACAGATTCAATCTCGTGGATGGACGTTCAACATTGAGGAAGGCATAACGCTACTACCTGAt                 <  1:219609/301‑1 (MQ=255)`  
                                                                                                                                                                                                                                                                                           `gcCACCGCTGAGCAATCACTAGCATAACCCCTT‑GGGGCCTCTAAACGGGTCTTGAGGGGTTTTTTGCTGAAAGGAGGAACTATATGCGCTCATGCGATATGAACGTTGAGACTGCCGCTGAGTTATCAGCTGTGAACGACATTCTGGCGTCTATCGGTGAACCTCCGGTATCAACGCTGGAAGGTGACGATAACGCAGATGCAGCGAACGCTCGGCGTATTCTCAACAAGATTAACCGACAGATTAAATCTCGTGGATGGACGTTCAACATTGAggctgtctcttatacacatctccgagc               >  1:64044/1‑276 (MQ=255)`  
                                                                                                                                                                                                                                                                                           `attagagacaggCAATCACTAGCATAACCCCTG‑GGGGCCTCGAAACGGGTCTTGAGGGGTTTTTTGCTGAAAGGAGGTACTATATGCGCTCATGCGATATGAACGTTGAGACTGGCGCTGAGTAATCAGCTGTGAACGACATTCTGGCGTCTATCGGGGAACCTCCGGTATCAACGCTGGAAGGTGACGCTAACGCAGATGCAGCGAACGCTCGGCGTATTCTCAACAAGATTAACCGACAGATTCAATCTCGTGGATGGACGTTCAACATTGAGGAAGGCATAACGCTACTACCTGATGt               <  1:309067/290‑1 (MQ=255)`  
                                                                                                                                                                                                                                                                                               `ccGCTGAGCAATCACTAGCATAACCCCTT‑GGGGCCTCTAAACGGGTCTTGAGGGGTTTTTTGCTGAAAGGAGGAACTATATGCGCTCATGCGATATGAACGTTGAGACTGCCGCTGAGTTATCAGCTGTGAACGACATTCTGGCGTCTATCGGTGAACCTCCGGTATCAACGCTTGAAGGTGACGCTAACGCAGATGCAGCGAACGCTCGGCGTATTCTCAACAAGATTAACCGACAGATTCAATCTCGTGGATGGACGTTCAACATTGAGGAAGGCATAACGCTACTACCTGATGTTTAc           >  1:221500/1‑301 (MQ=255)`  
                                                                                                                                                                                                                                                                                                      `gCAATCACTAGCATAACCCCTT‑GGGGCCTCTAAACGGGTCTTGAGGGGTTTTTTGCTGAAAGGAGGAACTATATGCGCTCATGCGATATGAACGTTGAGACTGCCGCTGAGTTATCAGCTGTGAACGACATTCTGGAGTTTATCGGTGAACCTCCAGTATCAACGCTGGAAGGTGCCGCTAACGCAGATGCAGCGAACGCTCGGCGTATTCTCAACACGCTTAACCGACAGATTCAATCTCGTGGATGGACGTTCAACATTGAGGAAGGCAtgtcccttatcaccatctcttcgccaccgc    >  1:342447/1‑272 (MQ=255)`  
                                                                                                                                                                                                                                                                                                        `aaTCACTAGCATAACCCCTT‑GGGGCCTCTAAACGGGTCTTGAGGGGTTTTTTGCTGAAAGGAGGAACTATATGCGCTCATGCGATATGAACGTTGAGACTGCCGCTGAGTTATCAGCTGTGAACGACATTCTGGCGTCTATCGGTGAACCTCCGGTATCAACGCTGGAAGGTGACGCTAACGCAGATGCAGCGAACGCTCGGCGTATTCTCAACAAGATTAACCGACAGATTCAATATCGTGCATGGACGTTCAACATTGAGGAAGGCATAACGCTACTACCTGATGTTTACTCCacactg  >  1:138698/1‑296 (MQ=255)`  

                                                                                                                                                                                                                                                                                                           `|`                                                                                                                                                                                                                                                                                                            
`GTACTGTTAAGCTGCGTGACTTGGCTCTGGAGCGCGCTCGCCGTGCTAACTTCCAAGCGGACCAGATTATCGCTAAGTACGCAATGGGCCACGGTGGTCTTCGCCCAGAAGCTGCTGGTGCAGTGGTTTTCAAAGTGGAGTAATGCTGGGGGTGGCCTCAACGGTCGCTGCTAGTCCCGAAGAGGCGAGTGTTACTTCAACAGAAGAAACCTTAACGCCAGCACAGGAGGCCGCACGCACCCGCGCTGCTAACAAAGCCCGAAAGGAAGCTGAGTTGGCTGCTGCCACCGCTGAGCAATAACTAGCATAACCCCTT‑GGGGCCTCTAAACGGGTCTTGAGGGGTTTTTTGCTGAAAGGAGGAACTATATGCGCTCATACGATATGAACGTTGAGACTGCCGCTGAGTTATCAGCTGTGAACGACATTCTGGCGTCTATCGGTGAACCTCCGGTATCAACGCTGGAAGGTGACGCTAACGCAGATGCAGCGAACGCTCGGCGTATTCTCAACAAGATTAACCGACAGATTCAATCTCGTGGATGGACGTTCAACATTGAGGAAGGCATAACGCTACTACCTGATGTTTACTCCAACCTG  >  NC_001604.1/23862‑24458`

  

Alignment Legend

Aligned base mismatch/match (shaded by quality score): `ATCG/ATCG` `< 3 ≤` `ATCG/ATCG` `< 10 ≤` `ATCG/ATCG` `< 22 ≤` `ATCG/ATCG` `< 34 ≤` `ATCG/ATCG` `< 38 ≤` `ATCG/ATCG`

Unaligned base: `atcg`    Masked matching base: `atcg`    Alignment gap: `‑`    Deleted base: `‑`
